# Tumor Necrosis Factor Inhibition and Parkinson Disease: A Mendelian Randomization Study

**DOI:** 10.1212/WNL.0000000000011630

**Published:** 2021-03-23

**Authors:** Xiaoying Kang, Alexander Ploner, Nancy L. Pedersen, Sara Bandres-Ciga, Alastair J. Noyce, Karin Wirdefeldt, Dylan M. Williams

**Affiliations:** From the Departments of Medical Epidemiology and Biostatistics (X.K., A.P., N.L.P., K.W., D.M.W.) and Clinical Neuroscience (K.W.), Karolinska Institutet, Stockholm, Sweden; Laboratory of Neurogenetics (S.B.-C.), National Institute on Aging, National Institutes of Health, Bethesda, MD; Instituto de Investigación Biosanitaria de Granada (S.B.-C.), Spain; Preventive Neurology Unit (A.J.N.), Wolfson Institute of Preventive Medicine, Queen Mary University of London; Department of Clinical and Movement Neurosciences (A.J.N.), UCL Institute of Neurology, London; and MRC Unit for Lifelong Health and Ageing (D.M.W.), University College London, UK.

## Abstract

**Objective:**

To evaluate the effects of long-term tumor necrosis factor (TNF) inhibition on the risk and age at onset of Parkinson disease (PD), we performed a 2-sample Mendelian randomization study using genome-wide association studies (GWAS) summary statistics.

**Methods:**

Genetic variants in the vicinity of *TNFRSF1A*, the gene encoding TNF receptor 1 (TNFR1), were identified as predictive of pharmacologic blockade of TNFR1 signaling by anti-TNF therapy, based on genetic associations with lower circulating C-reactive protein (CRP; GWAS n = 204,402). The effects of TNF-TNFR1 inhibition were estimated for PD risk (n_cases_/_controls_ = 37,688/981,372) and age at PD onset (n = 28,568) using GWAS data from the International Parkinson's Disease Genomics Consortium and 23andMe, Inc. To validate variants as proxies of long-term anti-TNF treatment, we also assessed whether variant associations reflected anticipated effects of TNFR1 inhibition on Crohn disease, ulcerative colitis, and multiple sclerosis risk (n = 38,589-45,975).

**Results:**

TNF-TNFR1 signaling inhibition was not estimated to affect PD risk (odds ratio [OR] per 10% lower circulating CRP = 0.99; 95% confidence interval [CI] 0.91–1.08) or age at onset (0.13 years later onset; 95% CI −0.66 to 0.92). In contrast, genetically indexed TNF-TNFR1 signaling blockade predicted reduced risk of Crohn disease (OR 0.75; 95% CI 0.65–0.86) and ulcerative colitis (OR 0.84; 95% CI 0.74–0.97) and increased multiple sclerosis risk (OR 1.57; 95% CI 1.36–1.81). Findings were consistent across models using different genetic instruments and Mendelian randomization estimators.

**Conclusions:**

Our findings do not imply that TNF-TNFR1 signaling inhibition will prevent or delay PD onset.

**Classification of Evidence:**

This study provides Class II evidence that TNF-TNFR1 signaling inhibition is not associated with the risk or age at onset of PD.

Inflammation in the periphery is hypothesized to contribute to the pathogenesis of Parkinson disease (PD), particularly when present in the gastrointestinal tract.^1^ Tumor necrosis factor (TNF) is a potent proinflammatory cytokine that exerts a variety of biological effects by binding to its 2 receptors, triggering intracellular signaling.^2^ Several types of TNF inhibitor have been licensed for the treatment of systemic inflammatory disorders, including inflammatory bowel disease (IBD).^3^ In a recent large cohort study of patients with IBD, exposure to anti-TNF therapeutics was associated with lower PD risk, implying that TNF inhibition may be neuroprotective for PD.^4^ However, these findings were observational, and hence prone to several forms of bias that preclude a causal interpretation, including confounding and reverse causation. Moreover, results obtained from a sample of patients with IBD may not be generalizable to individuals without IBD.

Mendelian randomization is a valuable approach to help answer questions in pharmacoepidemiology.^5^ In Mendelian randomization studies relating to pharmacologic exposures, genetic variants that perturb the expression or function of a drug's target (often a specific protein) are used to anticipate the effects of modulating this target with therapeutic use.^6^ Due to Mendelian randomization principles, associations of this genetic variation with diseases are not expected to have arisen from confounding or reverse causation, thus aiding causal inference.^7^ In this study, we used a Mendelian randomization design to further evaluate the therapeutic potential of targeting TNF signaling for PD prevention or treatment, leveraging genetic variation in or near the gene encoding TNF receptor 1 (TNFR1) that is indicative of the blockade of proinflammatory TNF signaling via this receptor.^2^

## Methods

### Study Overview

**Figure 1:**
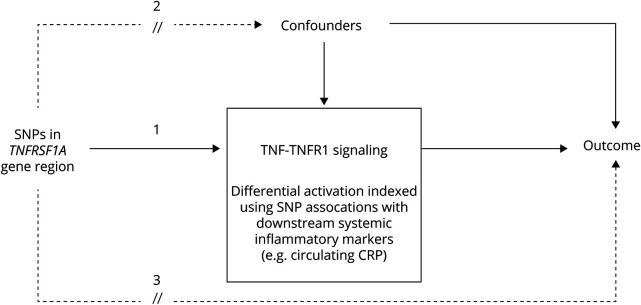
Overview of the Study Design By using only *cis*-acting variants in *TNFRSF1A*, the gene encoding tumor necrosis factor (TNF) receptor 1 (TNFR1), and modeling genetic associations with circulating inflammatory markers, the selected genetic instruments are expected to index the overall (averaged) long-term effect of blocking TNF-TNFR1 signaling on Parkinson disease and positive control outcomes (denoted by pathway 1). This is analogous to the pharmacologic modulation of TNF-TNFR1 signaling by TNF inhibitors in vivo (one effect of such an administration being a reduction in systemic inflammation). However, whereas observational studies of associations between TNF inhibitor use and outcomes may be confounded, we do not expect genetic variants within the gene region to be associated with confounders due to the random assignment of alleles at conception (i.e., pathway 2 should not be present). Moreover, variants in a specific gene region are likely to affect outcomes only through effects on the function or expression of the protein encoded by the gene, and hence variants should not be related to outcomes by other routes (pathway 3), which would bias our results. CRP = C-reactive protein; SNP = single nucleotide polymorphism.

This study used a 2-sample Mendelian randomization design (figure 1).^8^ We focused on single nucleotide polymorphisms (SNPs) in the vicinity of the gene *TNFRSF1A*, which encodes TNFR1, the principal effector of proinflammatory signaling following TNF agonism.^2^ We determined the extent to which genetic variation in this gene region is indicative of long-term TNF signaling blockade, based on associations of the regional SNPs with circulating markers of systemic inflammation reported by large genome-wide association studies (GWAS) of C-reactive protein (CRP) and cell count measures.^9,10^ We then combined data on selected variants with corresponding association statistics from GWAS of PD traits to test the effects of TNF-TNFR1 signaling inhibition on (1) the risk of PD, measured as self-reported or clinically ascertained disease status; and (2) age at PD onset, measured as self-reported age at motor symptoms manifestation, or age at PD diagnosis when the former was unavailable.^11,12^ This study provides Class II evidence that TNF-TNFR1 signaling inhibition is not associated with PD risk or age at onset, according to criteria of the American Academy of Neurology.

### Indexing TNF Inhibition

TNF signaling occurs through the binding of the cytokine to its 2 receptors, TNFR1 and TNFR2. We focused on indexing TNF-TNFR1 signaling specifically because agonism of TNFR1 mediates the proapoptotic and inflammatory effects of TNF (and hence the efficacy of anti-TNF therapeutics in the treatment of autoimmune diseases), whereas TNFR2 signaling contributes to tissue repair and neuron survival.^2,13,14^ Therefore, researchers have investigated the development of novel therapeutics to selectively antagonize TNFR1 without inhibiting TNFR2-mediated signaling.^15,16^ An effective response to anti-TNF treatment leads to lower systemic inflammation, reflected by reductions in circulating inflammatory biomarkers, such as CRP.^17^ Thus, to validate whether SNPs in the vicinity of *TNFRSF1A* index the modulation of proinflammatory TNF-TNFR1 signaling, we extracted data on associations of these variants with circulating CRP from a large GWAS meta-analysis (n = 204,402; table 1).^9^ To ensure that the CRP associations were not chance findings, we also examined associations of the SNPs with 2 cell count markers of inflammation—white blood cell count (WBC) and mean platelet volume (MPV)—from independent GWAS data (table 1).^10,18^

**Table 1:**
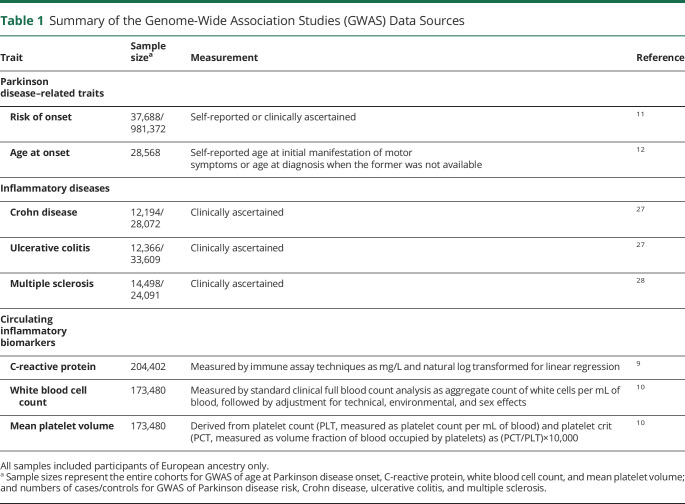
Summary of the Genome-Wide Association Studies (GWAS) Data Sources

In total, 23 SNPs were selected solely from within the gene's genomic coordinates and a narrow flank in either direction (chromosome 12; base pairs 6,437,923–6,451,280 ± 1 kb as per GRCh37 assembly). The choice of a narrow flanking region was adopted to minimize the possibility that the selected variants might associate with PD traits via pathways other than TNF-TNFR1 signaling, given that *TNFRSF1A* is located close to genes that encode other proteins with known immune-related roles, such as lymphotoxin β receptor gene *LTBR* (chromosome 12; base pairs 6,484,534–6,500,737 as per GRCh37 assembly).

### PD Data

Genetic association data for PD risk were derived from a meta-analysis of 16 case–control samples from the International Parkinson's Disease Genomics Consortium (IPDGC) and 23andMe, using the same protocol as adopted in a recent GWAS.^11^ This yielded a sample of 37,688 cases and 981,372 controls. Genetic association data for age at PD onset were based on a GWAS comprising 28,568 PD cases from a subset of the cases sampled by the IPDGC and 23andMe, in which the mean age at onset was 61.7 years (range 20–97).^12^ These samples are described further in table 1.

In 2-sample Mendelian randomization, participant overlap between the SNP exposure and SNP outcome samples may bias findings.^19^ However, sample overlap is likely to be nominal in this study because the exposure and outcome GWAS were conducted with largely independent samples: samples for CRP and cell count GWAS were derived primarily from population-based cohorts assembled by the Cohorts for Heart and Aging Research in Genomic Epidemiology (CHARGE) consortium,^9^ and PD GWAS samples were derived mainly from independent case–control studies assembled by the IPDGC and the 23andMe user base (table 1).^11^

### Positive Control Analyses

To validate our study design, we conducted positive control analyses using risk of Crohn disease, ulcerative colitis, and multiple sclerosis as additional outcome traits in our Mendelian randomization models. Protective effects of variants indexing TNF-TNFR1 signaling inhibition were expected for Crohn disease and ulcerative colitis risk because anti-TNF therapies have been approved for treating the 2 conditions.^20^ We anticipated a detrimental effect of variants indexing TNF-TNFR1 signaling inhibition on multiple sclerosis, given that the risk of multiple sclerosis is increased by anti-TNF treatment among patients with other autoimmune conditions, and symptom exacerbation has been reported by trials of anti-TNF therapies as treatments for multiple sclerosis.^21–26^ For analyses of these positive control outcomes, we used publicly available GWAS summary statistics with overall sample sizes ranging from 38,589 to 45,975 (table 1).^27,28^

### Statistics

Prior to statistical analyses, summary statistics for the associations of *TNFRSF1A* variants with CRP and outcomes were harmonized by aligning the coding of association statistics to the same reference allele (table 2). SNPs were excluded if these were not present in both CRP and outcome datasets, or where the coding of SNPs was ambiguous (palindromic SNPs with minor allele frequencies over 0.4).

**Table 2:**
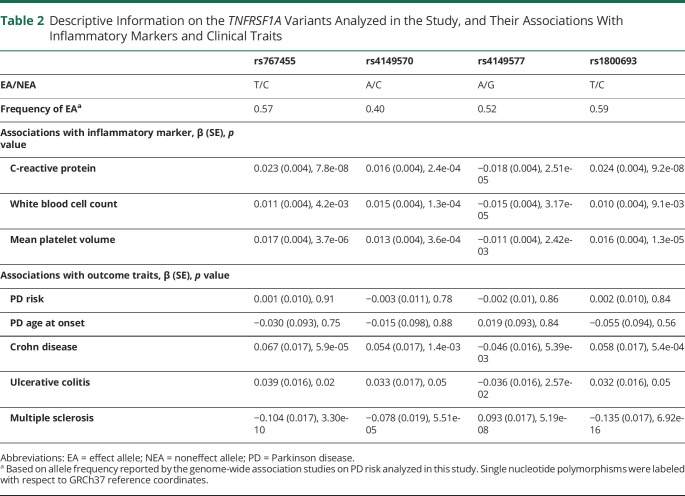
Descriptive Information on the *TNFRSF1A* Variants Analyzed in the Study, and Their Associations With Inflammatory Markers and Clinical Traits

We conducted Mendelian randomization models based on 3 approaches. In the primary analysis, we applied conservative linkage disequilibrium (LD) clumping (*r*^*2*^ < 0.001) to the set of SNPs in the region with CRP association *p* values under 0.05, to select independent SNPs with the strongest evidence for association with systemic inflammation. Mendelian randomization results based on this selection criterion were then obtained using Wald estimation, given that a single SNP (rs767455) was retained for analyses. Mendelian randomization estimates can be biased when the genetic variants used in analysis are weak instruments for the exposure being indexed.^29^ Thus, to indicate the strength of this SNP as an instrumental variable, the *F* statistic for its association with CRP was estimated from the *F* distribution based on the *p* value and sample size of SNP–CRP association, with 1 degree of freedom.

For secondary analyses, we aimed to retain more variants—which may explain more variance in CRP and therefore potentially improve the power of Mendelian randomization models—by including all regional SNPs after liberal LD clumping (*r*^2^ < 0.8). The clumped SNPs were then filtered by requiring false discovery rate–corrected CRP associations with *p* values under 0.05, and further associations of the variants with both WBC and MPV (using an unadjusted *p* threshold <0.05), ensuring consistent and robust associations of variants with inflammatory signaling were retained. Next, Mendelian randomization estimates based on the set of retained SNPs were calculated using an extension to the inverse variance weighting (IVW) method with principal components to account for the residual correlations between selected SNPs.^30^ This method relies on reference data to adjust for estimated correlations between the SNPs, for which we used data on 502 individuals of European ancestry of the 1000 Genomes project, phase 3.^31^ Principal components explaining over 99% of variance in the weighted correlation matrix were used in the IVW estimator.

Third, after examining the functional annotation of SNPs in the gene region, we also assessed specific associations of a single SNP (rs1800693) with traits, since this SNP is suggested to affect alternative splicing of the *TNFRSF1A* transcript to create a soluble TNFR1 isoform, which acts similarly to anti-TNF therapeutics.^32^ In this application, we evaluated genotype-trait associations for the functional SNP rs1800693—i.e., not weighted by CRP difference—and results were expressed per copy of the CRP-lowering allele (denoted in the Results as “TNF-inhibiting allele”).

All Mendelian randomization outputs were scaled to be expressed per 10% reduction in circulating CRP, indicative of the direction of effect anticipated from TNF inhibition.^33^ In plots, outputs from logistic regression models were presented as log-odds to scale appropriately alongside βs from linear regression models. Corresponding odds ratios (ORs) and 95% confidence intervals (CIs) for binary outcomes are reported in the Results. Given that we had strong priors for non-null results from positive control analyses, we did not adjust these results for multiple testing correction.

All analyses were performed in R version 3.6.1 (2019-07-05) using *TwoSampleMR* and *mendelianRandomization* packages.^31,34^

### Standard Protocol Approvals, Registrations, and Patient Consents

For each GWAS study included in the present work, written informed consent was obtained from all participants and the studies were approved by relevant ethics review boards. Because our study was based on existing summary-level genetic results—i.e., without accessing individual-level genetic data—no additional ethical approval was required.

### Data Availability

The majority of genetic association statistics used in this study are presented in table 2. Full CRP GWAS summary statistics can be obtained by contacting the corresponding authors for the CHARGE Inflammation Working Group.^9^ Summary statistics from GWAS of WBC and MPV are publicly available at bloodcellgenetics.org/.^35^ Full PD GWAS data can be obtained via research project applications to 23andMe, Inc. and the IPDGC.

## Results

**Figure 2:**
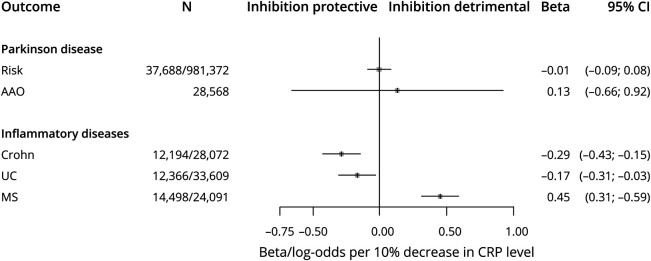
Estimated Effects of Tumor Necrosis Factor (TNF)–TNF Receptor 1 (TNFR1) Inhibition on Outcomes From Mendelian Randomization Models Using Single Nucleotide Polymorphism rs767455 as a Genetic Instrument AAO = age at onset; CI = confidence interval; Crohn = Crohn disease; CRP = C-reactive protein; MS = multiple sclerosis; UC = ulcerative colitis.

A total of 23 SNPs in the vicinity of *TNFRSF1A* were present in the CRP data. One SNP in the region—rs767455—was retained following conservative LD clumping (table 2). This exonic, synonymous SNP has a minor allele frequency of approximately 0.4 and had the strongest evidence for association with CRP of SNPs in the region (*p =* 7.8 × 10^−8^). It is in high LD with the presumed functional variant (rs1800693) in this region (*r*^2^ = 0.84)^32^ and the variant is also associated with circulating WBC (*p =* 4.2 × 10^−3^) and MPV (*p =* 3.7 × 10^−6^), with consistent directions for all 3 inflammatory markers. Using this variant in Mendelian randomization models, no effects of TNF-TNFR1 inhibition on PD risk (OR 0.99 per 10% reduction in circulating CRP; 95% CI 0.91–1.08) or age at onset (0.13 years of increase; 95% CI −0.66 to 0.92) were predicted (figure 2). In contrast, genetically indexed inhibition of TNF-TNFR1 signaling was predicted to reduce the risk of Crohn disease (OR 0.75; 95% CI 0.65–0.86) and ulcerative colitis (OR 0.84; 95% CI 0.74–0.97) and increase the risk of multiple sclerosis (OR 1.57; 95% CI 1.36–1.81), as expected. The estimated *F* statistic for the association of rs767455 with CRP was 28.9, suggesting that these Mendelian randomization models were unlikely to have been affected by weak instrument bias (values of *F* under 10 are of concern).

**Figure 3:**
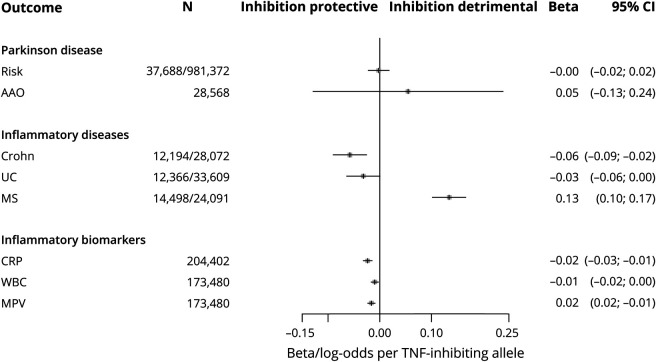
Associations of rs1800693 Genotype With Various Traits Differences in traits are per copy of tumor necrosis factor (TNF)–inhibiting allele, i.e., the allele associated with lower circulating C-reactive protein (CRP). AAO = age at onset; CI = confidence interval; Crohn = Crohn disease; MPV = mean platelet volume; MS = multiple sclerosis; UC = ulcerative colitis; WBC = white blood cell count.

In secondary analyses, the more liberal LD clumping yielded 4 correlated SNPs associated with CRP at false discovery rate–corrected *p* < 0.05. Among these 4 SNPs, one was not associated with either WBC (*p* = 0.7) or MPV (*p* = 0.95) and was therefore removed, leaving 3 variants that were associated with all 3 inflammatory markers for secondary Mendelian randomization models (table 2). Mendelian randomization estimates based on the 3 SNPs, using adapted IVW methodology to account for correlations between the variants, were consistent with the main results: neither PD risk (OR 0.99; 95% CI 0.91–1.08) nor age at onset (0.14 years of increase; 95% CI −0.66 to 0.93) were predicted to be affected by TNF-TNFR1 blockade, whereas anticipated effects on other inflammatory diseases were observed (table 3). Similarly, no associations of the genotypes of implicated functional variant (rs1800693) with PD risk (OR 1.00 per TNF-inhibiting allele; 95% CI 0.98–1.02) or age at onset (0.05 years of increase per TNF-inhibiting allele; 95% CI −0.13 to 0.24) were observed, despite strong associations of this SNP with other inflammatory traits (figure 3).

**Table 3:**
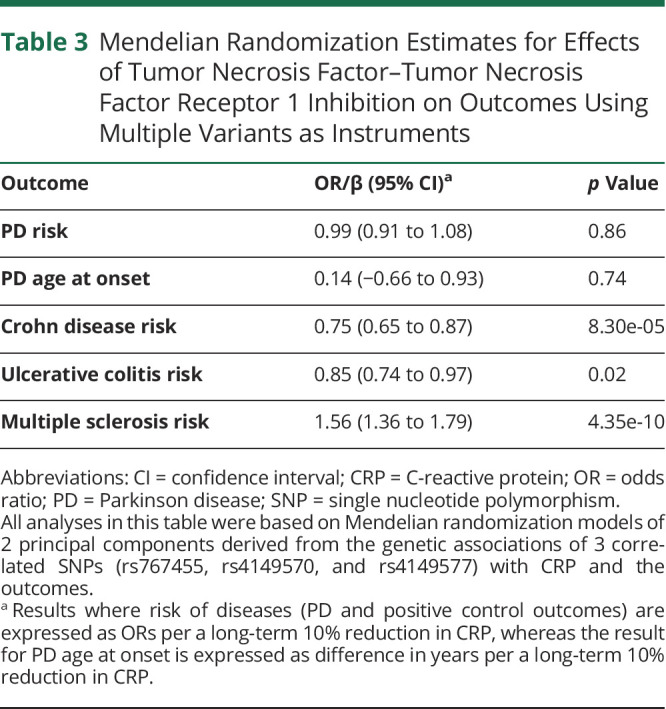
Mendelian Randomization Estimates for Effects of Tumor Necrosis Factor–Tumor Necrosis Factor Receptor 1 Inhibition on Outcomes Using Multiple Variants as Instruments

## Discussion

To our knowledge, this is the first Mendelian randomization study to address whether exposure to TNF inhibitors affects PD. Leveraging large genetic data on PD risk and age at PD onset, we found no evidence for the potential of TNF-TNFR1 signaling inhibition to prevent or delay PD onset in the general population.

By comparing patients with IBD with and without anti-TNF treatment in a cohort study, Peter et al.^4^ reported that exposure to TNF inhibitors was associated with a 78% decrease in PD risk (incidence rate ratio 0.22; 95% CI 0.05, 0.88). Although we showed no support for the potential of blocking TNF-TNFR1 signaling to lower PD risk, the results should be interpreted carefully in comparison to the observational study for several reasons. First, the 4 TNF inhibitors investigated by Peter et al.^4^—adalimumab, certolizumab, golimumab, and infliximab—all directly neutralize soluble or transmembrane TNF and disrupt both the TNFR1- and TNFR2-mediated pathways as well as other potential biological effects induced by TNF by any other pathways,^4,20,36^ whereas our results are specific to the selective inhibition of TNF-TNFR1 signaling transduction. However, we note that TNF-TNFR1 signaling is proposed as the major TNF-related neurotoxic pathway to intervene on for PD therapy.^14–16,37^ Second, given that IBD is rare, our findings are more relevant for the general population. The models test whether TNF-TNFR1 signaling blockade affects PD onset regardless of whether or not individuals have a background of severe systemic inflammation.^38^ Consequently, we do not exclude therapeutic benefits in individuals with certain underlying conditions, such as IBD. Third, Mendelian randomization estimates relate to an averaged effect of long-term exposure to the TNF-TNFR1 inhibitors across the life course; therefore, a null result may reflect either a true lack of an overall effect on PD risk at any stage of life or a sum of opposing, period-specific effects that balance out in our models. This scenario might be possible if, for instance, TNF-TNFR1 signaling inhibition is beneficial for PD in older age due to anti-inflammatory effects, but detrimental earlier in life for other reasons (e.g., increased susceptibility to infections or reduced neurodevelopment). Mendelian randomization estimates are also averaged across any tissue-specific effects, whereas therapeutic administration of TNF inhibitors may be limited to effects induced in the periphery. Fourth, because the 4 TNF inhibitors examined by Peter et al.^4^ are large molecules that do not cross the blood–brain barrier (when intact), they are assumed to mitigate peripheral inflammation principally, unless IBD has affected the integrity of the blood–brain barrier to allow TNF inhibitors to act in the cerebral parenchyma. However, the possibilities of opposing period- or tissue-specific effects of TNF inhibition on PD risk are speculative.

In addition to the risk of PD, we also considered whether TNF-TNFR1 signaling inhibition may affect the age at PD onset for its relevance to the course of disease progression in lieu of large GWAS of longitudinally measured PD progression, which are not currently available. For a progressive disease such as PD, an exposure affecting the age at onset could do so by either delaying the pathogenesis of PD (i.e., the disease is triggered later) or modifying the rate of PD progression once the disease process has commenced. In the present study, the long-term blockade of TNF-TNFR1 signaling did not affect the age at PD onset, implying limited potential for TNF inhibitors to affect PD progression (while keeping the aforementioned caveats about interpreting genetic vs pharmacologic results in mind).

The strengths of our study include the use of large-scale genetic data in a Mendelian randomization design to facilitate causal inference, robust metrics for the Mendelian randomization analyses (e.g., satisfactory instrument strength and functional relevance of the genetic variation), and positive control analyses that indicate the validity of using the chosen genetic variants to predict therapeutic effects. This study's main limitations are that we could not examine whether there is an interaction between inflammatory status and TNF inhibition on PD risk or index the effects of TNF inhibition on direct measures of PD progression.

In conclusion, our findings do not support the notion that long-term blockade of TNF-TNFR1 signaling will prevent or delay PD onset. Human genetics provides valuable guidance for therapeutic programs because the chances of successful drug development are substantially improved when genetic evidence links a drug's target to an indication for therapeutic use.^39^ Hence, our findings should be considered as part of discourse on the repurposing potential of TNF inhibitors for PD prevention and treatment, particularly if selective inhibitors of TNF-TNFR1 signaling become available. Future Mendelian randomization studies could use large-scale GWAS of PD progression to help evaluate the disease-modifying potential of selective TNFR1 inhibitors for PD treatment.

## Glossary

CHARGE: Cohorts for Heart and Aging Research in Genomic Epidemiology
CI: confidence interval
CRP: C-reactive protein
GWAS: genome-wide association studies
IBD: inflammatory bowel disease
IPDGC: International Parkinson's Disease Genomics Consortium
IVW: inverse variance weighting
LD: linkage disequilibrium
MPV: mean platelet volume
OR: odds ratio
PD: Parkinson disease
SNP: single nucleotide polymorphism
TNF: tumor necrosis factor
TNFR1: tumor necrosis factor receptor 1
WBC: white blood cell count
